# Long-term outcomes of heparin-induced thrombocytopenia after cardiac surgery

**DOI:** 10.1016/j.xjon.2024.10.029

**Published:** 2024-11-10

**Authors:** Emily Rodriguez, Maria Daskam, Benjamin L. Shou, Charles Woodrum, Ria Gupta, Kathryn E. Dane, Diane Alejo, Marc Sussman, Stefano Schena

**Affiliations:** a Division of Cardiac Surgery, The Johns Hopkins University School of Medicine, Baltimore, Md; b Department of Linguistics, The Ohio State University, Columbus, Ohio; c Department of Pharmacology, The Johns Hopkins Hospital, Baltimore, Md; d Division of Cardiothoracic Surgery, Medical College of Wisconsin, Milwaukee, Wis

**Keywords:** heparin, perioperative care, mortality, outcomes, thrombocytopenia, cardiac surgery, heparin-induced thrombocytopenia

## Abstract

**Objective:**

Heparin-induced thrombocytopenia (HIT) after cardiac surgery may lead to greater morbidity and mortality than predicted preoperatively. The aim of this study is to assess long-term outcomes of patients surviving HIT after cardiac surgery.

**Methods:**

Single-institution, retrospective study of adult patients who underwent cardiac surgery between 2011 and 2023 and developed HIT postoperatively. The institutional Society of Thoracic Surgeons database and electronic medical record were integrated with longitudinal data from phone questionnaires. HIT was defined by combined clinical (4Ts score) and serologic manifestations: a platelet decrease >50% from preoperative baseline, a high optical density positive heparin-PF4 antibody test, and a positive serotonin release assay.

**Results:**

We identified 88 of 11,658 patients (0.8%) with HIT after cardiac surgery. The majority were male (74%), white (73.8%), and with a mean age of 65.6 ± 11.6 years. Seventy-seven (87.5%) survived to discharge, had a 4Ts score of 5 [4-6], and 58 (75.3%) were discharged on oral anticoagulation, with only 22 (28.6%) receiving treatment for the past 3 months, for a median of 1.3 [0.8-4.5] years. Median length of stay was 24 [17-35] days and length of follow-up was 4.6 [0.3-12] years. Readmission occurred in 70.1% (n = 54) of patients, with an average of 3 [1-6] readmissions/patient. Causes of death during follow-up included cardiac (n = 7, 24.1%), infectious (n = 6, 20.7%), or neurologic events (n = 5, 17.3). Ten-year survival probability was 48%.

**Conclusions:**

Patients who develop HIT after cardiac surgery have an overall poor prognosis even after hospital discharge. In addition to prolonged hospitalization, patients experience further complications leading to frequent early readmissions and elevated mortality in the long-term.

**Figure:**
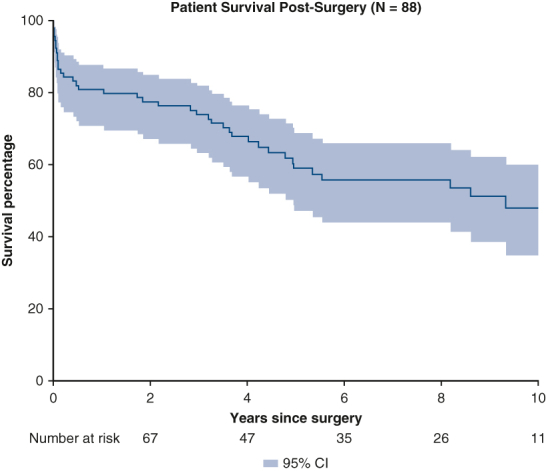
Long-term survival in patients who develop HIT and survive to discharge is <50%.

**Central Message**

Cardiac surgery patients who develop HIT postoperatively and survive, experience at least 1 HIT-related readmission within the first year and a 52% risk of mortality 10-years following surgery.

**Perspective**

HIT after cardiac surgery carries a stark prognosis, even after hospital discharge, with 52% long-term mortality rate, greater incidence of early readmission, and substantial ongoing complications. This study characterizes significant gaps in understanding long-term outcomes, emphasizing the need for closer post-discharge surveillance and possibly improved anticoagulation protocols.

Heparin-induced thrombocytopenia (HIT) is an immune-mediated and potentially lethal response to heparin administration leading to the formation of IgG antibodies directed against the heparin/platelet factor 4 (PF4) complex with consequent platelet activation, release of prothrombotic microparticles, platelet clumping, and thrombocytopenia.^1^^,^^2^ Heparin is largely used in cardiac surgery for prophylaxis and treatment of prothrombotic events (ie, atrial fibrillation, acute coronary syndromes, deep venous thrombosis, etc) and for institution of cardiopulmonary bypass (CPB).^3^ The estimated incidence of HIT is 1-2% among patients undergoing cardiac surgery, and the odds of developing HIT increase 10-fold in patients who receive heparin pre- or postoperatively.^2^^,^^4^ The diagnosis of HIT remains challenging because thrombocytopenia is commonly observed in the majority of patients after cardiac surgery. Confounding factors include platelet consumption, blood product administration, hemodilution, and a high rate of anti-PF4/heparin antibody seropositivity (“secondary thrombocytopenia”). Early detection of HIT depends on a combination of a high index of suspicion, risk stratification, history of recent exposure, as well as serologic detection of anti-PF4/heparin antibodies combined with positive result on the serotonin release assay.^5^

Patients who undergo cardiac surgery complicated by HIT may experience significantly greater risk of perioperative mortality and major complications including stroke, acute renal insufficiency, respiratory failure, and limb ischemia. Those who survive and are discharged may, in turn, face health challenges ranging from increased rate of readmission to shorter life expectancy, as well as increased health care costs.^6^ Because of the rarity of HIT and the high mortality associated with it, the fate of patients with HIT who survive to discharge has remained somewhat unexplored. The aim of this study is to characterize the long-term outcomes, such as delayed complications, incidence of readmission, and mortality, in patients who develop and survive HIT after cardiac surgery.

## Methods

### Study Population

This study was reviewed by the Johns Hopkins University Institutional Review Board (IRB00373654; approved September 5, 2023). We performed a query of our electronic medical records and institutional Society of Thoracic Surgeons (STS) database on cardiac surgery procedures using CPB between July 2011 and October 2023. Patients who underwent cardiac transplantation or received ventricular assist devices were excluded. Study patients were subsequently identified on the basis of a clinical and serologic diagnosis of HIT and survival to discharge. Patients were defined as HIT positive if they developed a platelet decrease >50% from preoperative baseline within 48 to 72 hours of the index procedure and had a positive anti-PF4 antibody screen confirmed by a subsequent positive serotonin release assay. Using severity of thrombocytopenia, timing of platelet count decrease in relation to previous heparin exposure, presence of thrombosis or other sequalae, and consideration for other probable causes for thrombocytopenia, 4Ts scores were calculated.^5^ Data from the STS database were supplemented with information obtained via manual chart review and phone interviews. All patients who participated in the phone interview provided informed consent.

### Statistical Analysis

Continuous variables were assessed for normality using the Kolmogorov-Smirnov test. Normally distributed variables were presented as mean +/− standard deviation. Non-normally distributed variables were presented as median with interquartile range.

To assess the frequency of complications postsurgery, we analyzed time to occurrence of vascular events, renal complications, pulmonary embolism, and stroke. Patients without a recorded complication were censored at date of last follow-up or death. Cumulative incidence was calculated using a stepwise function to visualize the proportion of patients who developed these complications. Each step increment corresponds to an event, normalized by the total number of patients at risk in the beginning of the study period. To analyze readmissions and complications postsurgery, a time-to-event analysis with a Nelson-Aalen fitter was used to estimate the cumulative hazard of complications as a function of time, with patients being censored at last follow-up or death. A competing risk with repeating events analysis was used to assess complications, accounting for the burden of multiple complications of the same type on the same patient and censoring from the competing risk of death. All analyses were performed using Stata/SE 18 for Windows. Analysis for recurrent events was performed using the reliability Python package with the MCF nonparametric class and the lifelines Python package with the Nelson Aalen fitter class.^7^

## Results

Within the study period, we identified 11,658 adult patients who underwent cardiac surgery using CPB. A total of 88 patients (0.8%) were diagnosed with HIT postoperatively. Of these, 11 patients (12.5%) died during hospitalization. The baseline characteristics of the study population are reported in Table 1. The average STS predicted risk of mortality for the assessed procedures was 2.3 ± 1.4%. In this cohort, 74% (n = 66) were male, 73.8% (n = 65) were white, and the average age and body mass index were 65.6 ± 11.6 years and 31.0 ± 7.1 kg/m^2^, respectively. The most commonly performed procedures were coronary artery bypass graft surgery (CABG; 41.6%, n = 32), aortic valve replacement with concomitant CABG (10.4%, n = 8), and isolated aortic valve replacement (7.8%, n = 6). Average CPB and crossclamp times were 146.5 ± 70.5 and 94.9 ± 47.7 minutes, respectively (Table 2).

**Table 1:** Baseline characteristics of patients who developed heparin-induced thrombocytopenia after cardiac surgery (N = 88)

Baseline characteristics	Mean ± SD or n (%)
Mean age, y	65.6 ± 11.6
Female sex	22 (25.0)
Race	
White	65 (73.8)
Black	13 (14.8)
Asian	4 (4.5)
Other	3 (3.4)
Diabetes mellitus	35 (39.8)
Hypertension	66 (75.0)
Body mass index, kg/m^2^	31.0 ± 7.1
Peripheral artery disease	7 (7.9)
Obesity	44 (50.0)
Renal failure requiring dialysis	2 (2.3)
Redo cardiac surgery	5 (5.7)
Previous exposure to heparin	54 (61.4)
Inpatient mortality	11 (12.5)

**Table 2:** Peri- and postoperative features of patients surviving heparin-induced thrombocytopenia (n = 77)

Procedure details	n (%)
Surgery type	
Isolated CABG	32 (41.6)
AV replacement + CABG	8 (10.4)
AV replacement	6 (7.8)
MV replacement	3 (3.9)
MV repair	3 (3.9)
MV replacement + CABG	3 (3.9)
AV replacement + MV replacement	1 (1.3)
Other	21 (27.3)
Mean CPB time, min	146.5 ± 70.5
Mean crossclamp time, min	94.9 ± 47.7
Mean STS-PROM∗	2.3 ± 1.4%
Median length of stay, d [IQR]	24.0 [17.0-35.0]
Mean length of follow-up, y	0.3-12.0
Median 4Ts score	5 [4-6]
Median high-dose SRA [IQR]	89.0 [74.5-100.0]
Median low-dose SRA [IQR]	0.0 [0.0-3.0]
Mean PF4 OD units	1.95 ± 1.01
Median baseline platelet count, ×10^3^ [IQR]	190 [156-219]
Median platelet nadir, ×10^3^ [IQR]	58 [40.5-75.5]
Anticoagulation upon discharge	
Warfarin	52 (67.5)
Direct oral anticoagulants	5 (6.5)
Other	3 (3.9)
None	17 (22.1)
Discharge location	
Transitional care unit/rehabilitation center	24 (31.2)
Home	48 (62.3)
Nursing home	1 (1.3)
Other	4 (5.2)

*CABG*, Coronary artery bypass grafting; *AV*, aortic valve; *MV*, mitral valve; *CPB*, cardiopulmonary bypass; *STS-PROM*, Society of Thoracic Surgeons predicted risk of mortality; *IQR*, interquartile range; *SRA*, serotonin release assay; *PF4*, platelet factor 4; *OD*, optical density.

∗ Mean STS-PROM only applies to cases eligible for STS risk score calculation.

Patients had a baseline median platelet count of 190,000 IQR: [156,000-219,000] and postoperative nadir of 58,000 [40,500-75,500]. Upon recognition of thrombocytopenia, 18% of patients were also diagnosed with thrombosis. Median calculated 4Ts score was 5 [4-6] and average PF4 antibody assay optical density units were 1.95 ± 1.01. Upon suspicion for HIT, heparin administration was discontinued, and patients were mostly transitioned to an intravenous bivalirudin infusion as bridge therapy to oral anticoagulation with warfarin. At discharge, 60 (77.9%) patients were prescribed anticoagulation. Of the remaining 17 (22.1%) patients, 6 (7.8%) were left untreated as the result of major inpatient bleeding events (eg, intracranial hemorrhage, gastrointestinal bleed). Lack of anticoagulation therapy at discharge could not be determined for 11 patients. Of those discharged with anticoagulation, 52 (67.5%) received warfarin, whereas 8 (10.4%) patients were prescribed direct oral anticoagulants or other anticoagulants. Among those managed with warfarin, 42 (54.5%) patients required long-term anticoagulation for reasons other than HIT (eg, atrial fibrillation, prosthetic mechanical valve, etc), and 29 patients had treatment indications specifically for HIT. Of these last 29 patients, 22 (28.6%) were maintained on oral anticoagulation beyond 3 months, for a median of 1.3 [0.8-4.5] years (Table 2).

**Figure 1:**
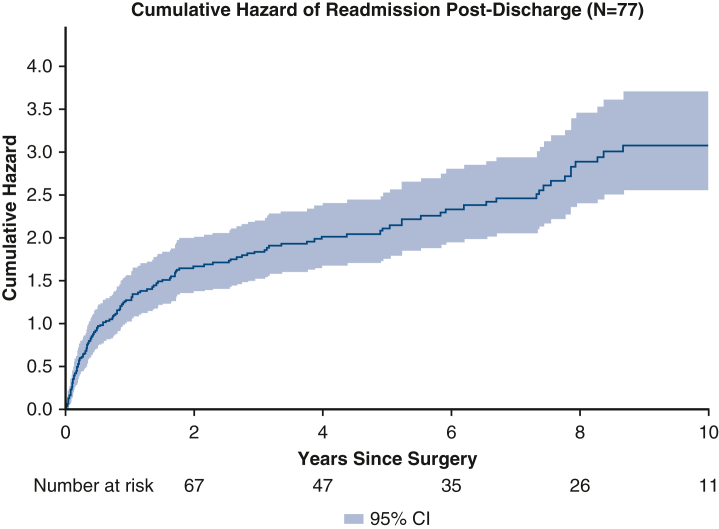
Cumulative hazard for readmissions in patients who develop heparin-induced thrombocytopenia after cardiac surgery and survive to discharge. X-axis is the years since surgery, and the y-axis is the cumulative hazard of readmissions expected for a patient. *Blue shading* shows the 95% confidence interval.

Median index hospital length of stay was 24 [17-35] days, the length of follow-up for the cohort was 4.6 [0.3-12] years, and the follow-up completion rate was 100%. Average number of readmissions was 3 [1-6] per patient. Patients were most often discharged home (62.3%) or to a transitional care unit (31.2%, Table 2). Readmission occurred in 70.1% (n = 54) of patients. More than one half of these patients presented to the hospital within 1.4 years of discharge. Using repeating events analysis, the cumulative hazard of readmission was 1.3 at 1 year, 2.1 at 5 years, and 3.1 at 10 years after surgery (Figure 1).

**Figure 2:**
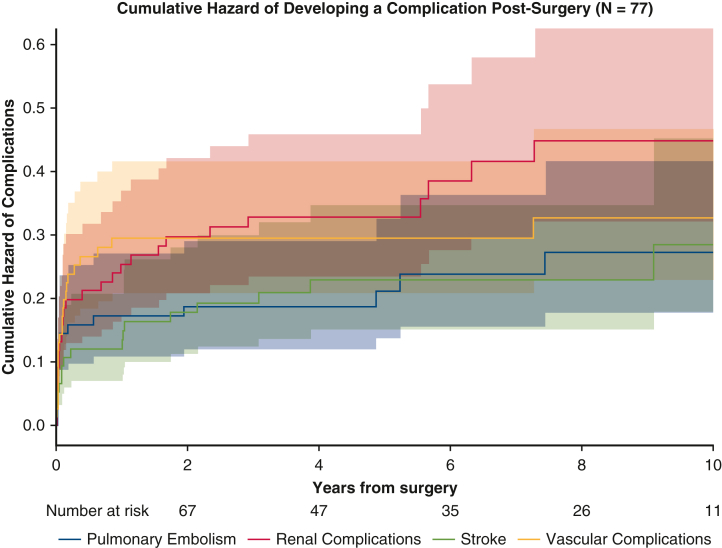
Competing risks curve for time to onset of significant postdischarge complications in patients after surgery. Each is complication shown with respect to the competing risk of mortality death and the burden of multiple complications of the same type on the same patient as a mean cumulative complication post-surgery. Complications include vascular issues (deep vein thrombosis, limb ischemia, gangrene), renal complications (acute kidney injury, acute renal failure, progressed chronic kidney disease), pulmonary embolism, and stroke. The x-axis represents years from surgery and the y-axis represents the cumulative hazard expected for a patient for each complication. 95% confidence interval depicted by *shading*.

In 74.0% (n = 57) of patients, we observed at least 1 new prothrombotic complication evolving from their initial HIT clinical manifestation, including stroke, pulmonary embolism (PE), new or progressed renal insufficiency, and new vascular events (Table 3). Within the first year after surgery, there were 9 instances of stroke, 19 PEs, 22 new renal complications, and 22 new vascular complications. From surgery to the 5-year timepoint, there were 16 strokes, 15 PEs, 24 new renal complications, and 22 new vascular complications amongst 41 patients (Table 3). Accounting for the competing risk of death, the cumulative hazard 4 years after surgery for developing PE, stroke, renal, and vascular complications was 0.19, 0.23, 0.33, and 0.29, respectively (Figure 2).

**Table 3:** Incidences of complications after surgery in patients who developed heparin-induced thrombocytopenia after cardiac surgery

Complications (number of patients∗)	1 y (n = 77)	2 y (n = 77)	5 y (n = 62)	10 y (n = 41)
Stroke	9	13	16	17
Pulmonary embolism	13	14	15	17
Renal complications (acute kidney injury, progressed chronic kidney disease, acute renal failure)	19	22	24	28
Vascular complications (deep vein thrombosis, gangrene, limb ischemia)	22	22	22	23

∗ Number of patients at each time point include the total number of patients dead or still at risk for developing a complication at that time point.

**Figure 3:**
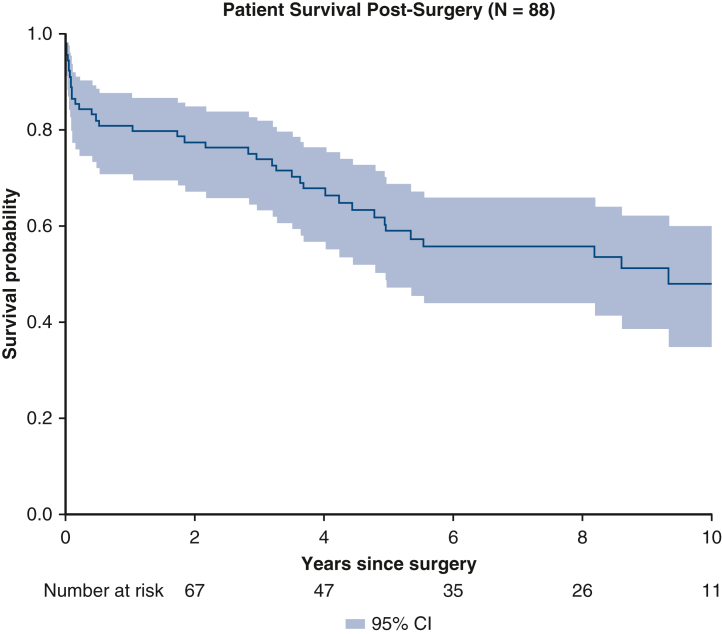
Kaplan-Meier survival probability for patients who developed heparin-induced thrombocytopenia and survived to discharge. *Blue shading* shows the 95% confidence interval.

In the overall cohort, the 1-, 5-, and 10-year survival probability was 80.7% (n = 71), 58.7% (n = 51), and 47.8% (n = 42) (Figure 3). Of those patients who survived to discharge, 37.7% (n = 29) died during follow-up. The main causes of death were cardiac (24.1%, n = 7), infectious (20.7%, n = 6), neurologic events (n = 5, 17.2%), other (n = 5, 17.2%), or unknown (n = 6, 20.7%) (Table 4).

**Table 4:** Cause of death in patients who developed heparin-induced thrombocytopenia after cardiac surgery and survived to discharge

Cause of death	n = 29
Cardiac	7 (24.1%)
Neurologic	5 (17.2%)
Infection	6 (20.7%)
Unknown	6 (20.7%)
Other	5 (17.2%)

## Discussion

In this single-institution study on long-term outcomes of patients who develop HIT after cardiac surgery and survive to discharge, we observed a substantial incidence of readmissions, HIT-related complications, and decline in survival over time. HIT is associated with longer length of stay, increased cost of the index admission, and greater mortality.^6^ Patients who develop HIT after cardiac surgery have an overall poor prognosis with mortality as high as 33%.^8^^,^^9^ A previous report of the National Inpatient Sample database suggested that operative mortality was approximately 11% in patients who developed HIT after cardiac surgery, which is similar to the 12.5% index hospitalization mortality rate we observed.^10^

The postoperative course in patients who develop HIT after cardiac surgery has been well described, but little is known regarding outcomes of patients after discharge.^8^^,^^11^, ^12^, ^13^, ^14^ Kerendi and colleagues^12^ reported that cardiac surgery patients who develop HIT are more likely to develop infectious complications, experience a greater rate of renal failure requiring hemodialysis, and a greater 30-day mortality (24.8%) compared with those without HIT.^11^ In a propensity-matched analysis, Brown and colleagues,^11^ showed that during index hospitalization, patients who underwent cardiac surgery and developed HIT had an operative mortality of 21.8% compared with 5.3% in patients without HIT. The authors also observed that thromboembolic events occurred in 29.1% of patients with HIT compared with 2.9% of patients without HIT.^11^ These observations were limited to a 30-day postoperative period.

This study aims to fill the current gap in knowledge regarding the fate of patients undergoing cardiac operations complicated with HIT by providing additional information on long-term outcomes, as well as morbidity and mortality risk beyond the immediate postoperative period. Besides confirming the elevated mortality during the index admission, for those who survived to discharge, we observed greater readmission rates, persisting complications, and incremental mortality in the long-term. In a study by Seigerman and colleagues,^10^ patients who developed HIT after cardiac surgery had a 50% increased risk of 30-day mortality. In several studies on long-term outcomes for patients undergoing CABG not complicated by HIT, the estimated 10-year survival ranges between 72% and 80%.^15^, ^16^, ^17^, ^18^ In contrast, our cohort 10-year survival for patients who developed HIT after isolated CABG was significantly lower at 62.5%.

We observed the largest decline in survival in the first 1.5 years post-discharge, suggesting that this early time frame may represent an especially critical period for close follow-up and management. The overall mortality rate of this cohort at the end of the follow-up period was 52.2%. Readmission rates among these patients were high, which suggests ongoing health challenges for those who develop HIT, with a resultant increase in resource use. Given that most readmissions occurred more than 30 days after discharge, this underscores both the prolonged nature of the downstream impact of HIT and the necessity for close monitoring beyond the immediate postdischarge period.

Previous literature has explored potential predictors for developing HIT. Associations with CPB time, body mass index, previous exposure to heparin, and preoperative thrombocytopenia have been investigated as potential contributors to developing HIT.^4^^,^^10^^,^^19^ In a recent study, we observed how CPB time has a significant association with HIT, but such variable was not an overall predictor of mortality or long-term development of complications after discharge.^4^ As previously reported, patients who developed major perioperative complications related to HIT were more likely to be discharged to a skilled facility and ultimately succumb to such complications.^10^

Acute peripheral vascular events were the most observed complication in the cohort, consisting of deep vein thrombosis, gangrene, and critical limb ischemia. These complications occurred earlier in the postdischarge period compared to other complications observed, with the greatest rate noted within the first 100 days post-discharge. This again reiterates how the initial period after hospital discharge may be a particularly vulnerable time for these patients. Interestingly, PE displayed a more delayed-onset pattern, with most of the cases emerging after 100 days from discharge. In contrast, renal complications exhibited a more gradual increase in incidence over time. At baseline, only 2 patients had preoperative kidney dysfunction requiring hemodialysis, but renal complications reached a peak incidence of more than 60% within the cohort by the end of the study period. The relationship between cardiac surgery and the subsequent development of acute kidney injuries in the perioperative period is well known, but this finding suggests that in patients who develop HIT, there is an additional risk of developing recurrent exacerbations eventually leading to renal failure. Collectively, these data highlight that many complications in patients who developed HIT after cardiac surgery occur within the first year after discharge. The sustained development of these complications, predominantly macro- or microvascular in nature, suggests that these patients need prolonged close monitoring beyond the immediate postoperative period, possibly with a long-term anticoagulation plan. Although we now have a better understanding of recurrent and more severe issues these complex patients may encounter, our study indicates the need for a more effective treatment strategy after discharge.

The initial medical treatment of postoperative HIT is still heavily based on high index of suspicion, where heparin administration is discontinued, and patients are promptly treated with nonheparin anticoagulants, such as fondaparinux, argatroban, or bivalirudin. Despite extensive anticoagulation, a significant proportion of patients were readmitted with new prothrombotic complications. The ongoing risk may persist as the result of continued immune system activation and long-term effects of thrombin generation. Such continued presence of circulating HIT antibodies can contribute to a persistent hypercoagulable state. The presence of these antibodies can lead to thrombosis even after discontinuing heparin administration, particularly if other triggers, such as surgery or inflammation, are present. Inadequate management of anticoagulation, particularly with warfarin, which demands consistent monitoring, along with ongoing endothelial damage from HIT, may also predispose patients to future thrombotic events. The American Academy of Physicians currently recommends that patients who develop HIT should be managed with continuous therapeutic oral anticoagulation for 3 months if they develop HIT with thrombosis.^20^ In line with the 2018 American Society of Hematology Guidelines recommendations, warfarin should not be administered until the platelet count rises above 150,000 and, in the initial acute period of HIT, warfarin is generally not recommended because of its initial reduction in protein C levels that can paradoxically increase thrombotic complications, including warfarin skin necrosis.^21^ In contrast, anticoagulants that directly inhibit thrombin, such as lepirudin or argatroban, are preferred during this critical period. Beyond this phase, warfarin is still extensively used in the mid- to long-term. In this frail population, the risks of bleeding and vascular complications associated with long-term warfarin therapy should be carefully considered. The delayed onset of the majority of the complications observed in our patient cohort suggest the presence of a persistent prothrombotic state that may benefit from a significantly longer anticoagulation therapy window than currently recommended, especially within the first-year post discharge.

Because of the low incidence of HIT and the high mortality associated with this complication, few studies have analyzed the long-term outcomes of patients who survive the index hospitalization. Further analysis of patients who were readmitted within 30 days or later suggests that early readmission is not a predictor of overall mortality. As observed in our study, the 10-year probability of survival in patients who develop HIT after cardiac surgery and survive to discharge is 40.3%. The Complications After Thrombocytopenia Caused by Heparin (CATCH) Registry^22^ includes more than 550 patients who have undergone cardiac surgery and tracks the incidence, management, and clinical consequences of heparin-associated thrombocytopenia. Through this registry, Lopes and colleagues^23^ showed that at 6 months, there was a 9% mortality rate in patients who survived to discharge. This rate is starkly greater than the one we observed, where 3.9% of patients died during the same observation period, thus highlighting the need for long-term data to better predict morbidity and mortality years after postoperative development of HIT. Causes of death outside of the inpatient period have yet to be reported, and the high percentage of patients in our cohort with an unknown cause of death reflects the lack of a defined clinical trajectory for these patients. Nevertheless, this study highlights that the development of HIT and associated complications may not necessarily lead to the ultimate cause of death but rather reflects the compounded morbidity associated with the onset of multiple complications over time.

The long-term complications described highlight the need for early diagnosis and treatment of HIT in patients who undergo cardiac surgery. Although the 4Ts score is widely used for predicting the risk of HIT, in the setting of relevant clinical and laboratory factors,^24^ this has been found to have low predictability for patients undergoing cardiac surgery, who inherently have another explanation for thrombocytopenia when placed on CPB.^5^^,^^21^^,^^25^^,^^26^ A modified 4Ts score has recently been developed that takes into consideration the use of mechanical circulatory support to help better risk stratify patients who develop thrombocytopenia after cardiac surgery.^26^ HIT is a relatively rare complication, and the potential co-existence of hereditary thrombophilias, such as factor V Leiden, should be considered. Lee and colleagues compared outcomes of patients with and without factor V Leiden in the setting of HIT and found a 9.7% incidence of factor V Leiden. In the same study, they found no difference in the incidence of venous or arterial thrombosis, PE, gangrene, stroke, or myocardial infarction between the groups, suggesting that factor V Leiden does not confer additional risks, and that the etiology of these deleterious complications seen in patients are attributable to HIT.^27^ In this cohort, the predominance of complications was likely driven by the immune-mediated effects of HIT; however, the possibility of underlying thrombophilias contributing to these events cannot be entirely ruled out.^28^ Screening for common hereditary thrombophilias could be beneficial in identifying patients at greater risk, allowing for tailored anticoagulation strategies and closer monitoring during follow-up. This consideration underscores the need for a comprehensive approach in managing patients with HIT, taking into account both acquired and genetic risk factors for thrombosis.

Prediction and prevention of the development of HIT after cardiac surgery remains difficult because of the multiple nonmodifiable risk factors present in the population undergoing cardiac surgery. Nevertheless, HIT occurrence remains rare yet excessively morbid. Treatment of postoperative HIT can be challenging and requires a multidisciplinary approach, including collaboration with caregivers and primary care physicians, because of the long-term sequelae that these patients may develop. Beyond the immediate, 30-day postoperative period, patients who develop HIT should have close follow-up for at least 1 year, with consideration for extended anticoagulation therapy if compatible with patients’ individual clinical status. After a year, shared decision-making and risk-benefit determination should guide future follow-up and prolonged anticoagulation need.

### Limitations

The presented study is characterized by a few limitations. First, given its retrospective nature of significantly relying on a combination of electronic medical record and phone-administered surveys, recollection bias and incompleteness of the data may limit data accuracy. We acknowledge that a major limitation of this study is that it is a single-arm investigation establishing a foundational literature for this understudied population. Future work with a propensity-matched analysis would enhance the comparative analysis of the HIT cohort and provide further insights. Since management and outcomes are determined on the basis of a single, tertiary academic center, the described findings may not be generalizable to other settings given differences in resources, individual practices, and specific demographics. Lastly, given that HIT is a relatively rare complication with a low incidence, the small sample size of the cohort may limit the statistical significance of associations and differences.

## Conclusions

Patients who develop HIT after cardiac surgery have an overall poor prognosis, even if they survive the initial diagnosis and are discharged from the hospital in stable clinical condition. This population invariably requires prolonged hospitalization, extensive physical rehabilitation, and numerous readmissions due to serious cardiovascular and infectious complications, also impacting healthcare costs. Although mortality may occur earlier in patients requiring rehospitalization, short-term outcomes do not predict long-term survival or the development of future complications. Increased surveillance within the first-year post-discharge, along with a prolonged anticoagulation plan during this period, may provide an opportunity to more effectively manage the long-term sequelae of postoperative HIT.

### Webcast

You can watch a Webcast of this AATS meeting presentation by going to: https://www.aats.org/resources/long-term-outcomes-of-heparin-7367.

**Figure:**
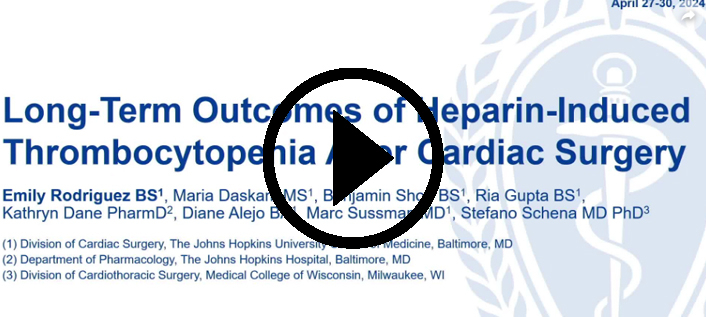

## Conflict of Interest Statement

The authors reported no conflicts of interest.

The *Journal* policy requires editors and reviewers to disclose conflicts of interest and to decline handling or reviewing manuscripts for which they may have a conflict of interest. The editors and reviewers of this article have no conflicts of interest.
